# Risk Factors for Pulmonary Tuberculosis in Patients with Lung Cancer: A Retrospective Cohort Study

**DOI:** 10.7150/jca.81616

**Published:** 2023-03-05

**Authors:** Kuang-Ming Liao, Chin-Chung Shu, Fu-Wen Liang, Yi-Chen Chen, Chia-Hung Yu, Jhi-Joung Wang, Chung-Han Ho

**Affiliations:** 1 Department of Internal Medicine, Chi Mei Medical Center, Chiali. Tainan, Taiwan; 2 Department of Internal Medicine, National Taiwan University Hospital, Taipei, Taiwan; 3 College of Medicine, National Taiwan University, Taipei, Taiwan; 4 Department of Public Health, College of Health Sciences, Kaohsiung Medical University, Kaohsiung, Taiwan; 5 Department of Medical Research, Kaohsiung Medical University Hospital, Kaohsiung, Taiwan; 6 Department of Medical Research, Chi Mei Medical Center. Tainan, Taiwan; 7 Department of Anaesthesiology, Chi Mei Medical Center, Tainan, Taiwan; 8 Cancer Center, Wan Fang Hospital, Taipei Medical University, Taipei, Taiwan; 9 Department of Information Management, Southern Taiwan University of Science and Technology, Tainan, Taiwan

**Keywords:** lung cancer, tuberculosis, risk factor, Taiwan Cancer Registry, population database

## Abstract

**Background**: Lung cancer increases the risk for pulmonary tuberculosis (PTB). The risk factors for newly diagnosed PTB are not known in lung cancer. This study analyzed risk factors of new-onset PTB among lung cancer patients in Taiwan.

**Methods**: Taiwan's National Health Insurance Research Database and Taiwan Cancer Registry were used to define PTB and lung cancer patients between 2007 and 2015. Considering that mortality was a competing risk event during the cancer treatment, Fine and Gray method was performed to estimate the possible risk factors for PTB among lung cancer patients.

**Results**: A total of 1,335 patients had PTB after lung cancer. The incidence of PTB increased with patients' raising age. Males had 1.7-fold (95% CI: 1.5-2.0) risk of PTB compared with females. Patients aged between 60-69 years (HR: 1.4; 95% CI: 1.1-1.8) and those ≥70 years (HR: 1.9; 95% CI: 1.5-2.4) had higher PTB risk than those aged under 50 years. Patients with history of pneumoconiosis and patients who received the treatments of surgery and chemotherapy also had significant increasing risk of PTB.

**Conclusion**: Screening for PTB may be important among lung cancer patients with the aforementioned risk factors.

## Introduction

Cancer is the second-leading cause of death worldwide and was responsible for approximately 9.6 million deaths in 2018^1^. Lung cancer is one of the most common cancers and the most common causes of cancer-related death globally 1. The immunosuppression status of cancer patients results in higher susceptibility to pulmonary tuberculosis (PTB) infection. Previous studies have shown that lung cancer increases the risk for PTB 2. A systematic review also revealed that active PTB was more common in patients with lung cancer and was most often observed in Asian males 3. The risk factors for PTB in lung cancer included previous PTB infection, chronic obstructive pulmonary disease, alcohol consumption, cigarette smoking, and/or diabetes mellitus 4. Taiwan has an intermediate PTB burden; the PTB prevalence was approximately 48-55 per 100,000 people in 2011-2014 5. The new-onset PTB incidence in patients with different types of malignancy had reported, including the mortality and recurrence rates among those patients 6. The risk factors for newly diagnosed PTB among lung cancer patients in Taiwan are not known. The aim of our study was to analyze risk factors for newly diagnosed PTB among patients with lung cancer in Taiwan using a population-based cancer registry database.

## Methods

### Data source

The Taiwan Cancer Registry (TCR) and Taiwan's National Health Insurance Research Database (NHIRD) from the Health and Welfare Data Science Center (HWDC) were used in this study. HWDC is an integrated data center with de-identified patient data that provides relevant information from health insurance claims for research purposes. The NHIRD is based on Taiwan's national health insurance program contains information about approximately 99% of all inpatient and outpatient medical benefit claims since 1996. The TCR is a population-based cancer registry database with cancer-related information since 1979. This study was conducted in compliance with the Declaration of Helsinki and was approved by the Research Ethics Committee of Chi Mei Hospital (IRB no. 10803-E01). The requirement for informed consent was waived due to the retrospective nature of the study.

### Patient selection and definition

In this cohort study, patients who enrolled in this study was following the below inclusion and exclusion criteria. The inclusion criteria was patients who were diagnosed with lung cancer were selected from the TCR between 2007 and 2015. Lung cancer diagnosis followed the International Classification of Diseases for Oncology, third edition (ICD-O-3: C34). To determine the new-onset PTB incidence after patients were diagnosed with lung cancer, the exclusion criteria was patients with a PTB history and enrolled patients without complete information about clinical stage, birth date, or sex. The follow-up duration began on the diagnosis date of cancer and ended on diagnosis date of PTB or December 31, 2017. PTB was identified using the International Classification of Diseases, Clinical Modification codes from inpatient and outpatient claims (ICD-9-CM: 010-012, 018; and ICD-10-CM: A150, A155, A154-A159) for treatment with PTB drugs, which were defined by Anatomical Therapeutic Chemical (ATC) classification codes.

**Figure 1:**
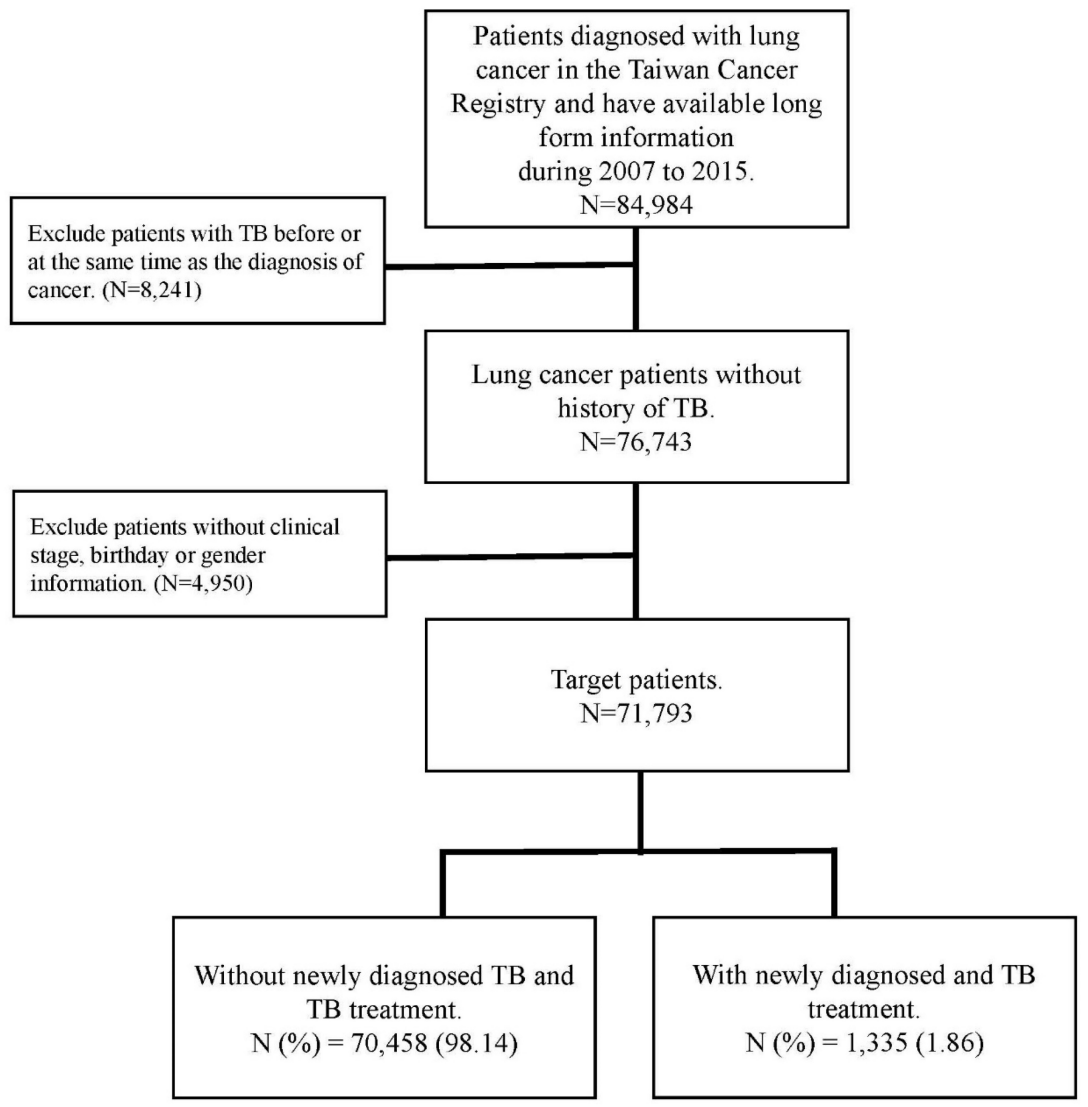
The flow chart of patient selection.

Patient age, sex, clinical stage of cancer, type of treatment, cell type, and smoking history were all considered confounding factors in this study. The comorbidities determined from medical claims records included diabetes (ICD-9-CM code: 250), chronic obstructive pulmonary disease (COPD) (ICD-9-CM code 490, 491,492,496), renal disease (ICD-9-CM code 582, 5831, 583.2, 583.3, 583.4, 583.5, 583.6, 583.7, 585, 586, 588.0), pneumoconiosis (ICD-9-CM 500, 502, 503, 505), hypertension (ICD-9-CM code 401-405), dyslipidemia (ICD-9-CM code 272.0-272.4), ischemic heart disease (ICD-9-CM code 410-414) and cerebrovascular disease (ICD-9-CM code 430-434). Those comorbidities were identified at least one year before the date of lung cancer diagnosis. The flowchart for patient selection is presented in Figure 1.

### Statistical analysis

The baseline information is presented as the frequency with percentage, and the differences between lung cancer patients with PTB and those without PTB were determined using the chi-square test. A Cox proportional hazards model was used to estimate the hazard ratios of PTB risk for each selected risk factor. To avoid violating the proportional hazards assumption, the Schoenfeld residuals approach was used to evaluate the assumption. Considering that mortality was a competing risk event during the cancer treatment, the Fine and Gray method 7, 8 was performed to compare the cumulative risk for PTB. Subgroup analysis was also performed for lung cancer patients with different cell type involvement. In addition, the cumulative incidence function of PTB was determined with Gray's test for comparing the PTB risk between different groups. SAS 9.4 (SAS Institute, Inc., Cary, NC, USA) was used to perform all statistical analyses. The curves of the cumulative incidence function were plotted using Stata 12 (Stata Corp, College Station, TX, USA). All significance levels were set at a p-value < 0.05.

## Results

A total of 71,793 lung cancer patients without a history of PTB were included. There were 1,335 patients who contracted PTB during the observation period. The crude incidence rate of new-onset PTB among lung cancer patients was 1.86%. The demographic information and the incidence rate of PTB in different characteristics of lung cancer patients show in Table 1.

Table 2 shows the risk factors for PTB in patients with lung cancer. Males had a 2.4-folds risk of PTB compared with females (adjusted HR, AHR: 2.4; 95% confidence interval, CI: 2.1-2.7). Patients aged between 60-69 years and those ≥70 years of age presented the higher risk of PTB (AHR: 1.5, 95% CI=1.2-1.9 and AHR: 2.2, 95% CI=1.8-2.8, respectively) than those aged <50 years. Compared with stage I lung cancer patients, patients had higher PTB risks in clinical stage II (AHR: 1.4, 95% CI=1.1-1.8), III (AHR: 1.52, 95% CI=1.2-1.9), and IV (AHR: 1.4, 95% CI=1.1-1.7).

Considering that mortality was a competing risk event during the cancer treatment, Table 3 shows the subdistribution hazard ratio of PTB to find the potential risk factor in lung cancer patients. Males still had a higher risk for PTB than females, with an adjusted HR of 1.7 (95% CI= 1.5-2.00). Patients aged between 60-69 years and those ≥70 years of age had also 1.4-fold risk for PTB (95% CI=1.1-1.8) and 1.9-fold risk for PTB (95% CI=1.5-2.4), respectively, than those aged <50 years. However, the clinical stage of cancer was not statistically significant for PTB risk in lung cancer patients. Moreover, patients who received surgery or chemotherapy, and those with pneumoconiosis and hypertension presented significant higher risk for PTB.

Table 4 shows the risk factors for PTB in patients with lung cancer involve different cell types. Of patients with non-small cell lung cancer, the traditional hazard ratio of PTB and the subdistribution hazard ratio which considering of competing mortality risk, sex, age, and patients with pneumoconiosis presented the similar results for statistically significant risk of PTB. However, the different results in clinical stage and treatment types. Considering of competing mortality risk, clinical stage did not be a risk factor of PTB, but lung cancer patients who received surgery and chemotherapy had higher risk for PTB than patients who did not receive surgery and chemotherapy, with adjusted HRs of 1.6 (95% CI=1.3-1.9) and 1.2 (95% CI=1.0-1.3), respectively. In small cell lung cancer, only patients who received chemotherapy had a higher risk for PTB than those without chemotherapy, with an adjusted HR of 2.4 (95% CI=1.4-4.4) after considering of competing mortality risk.

**Figure 2:**
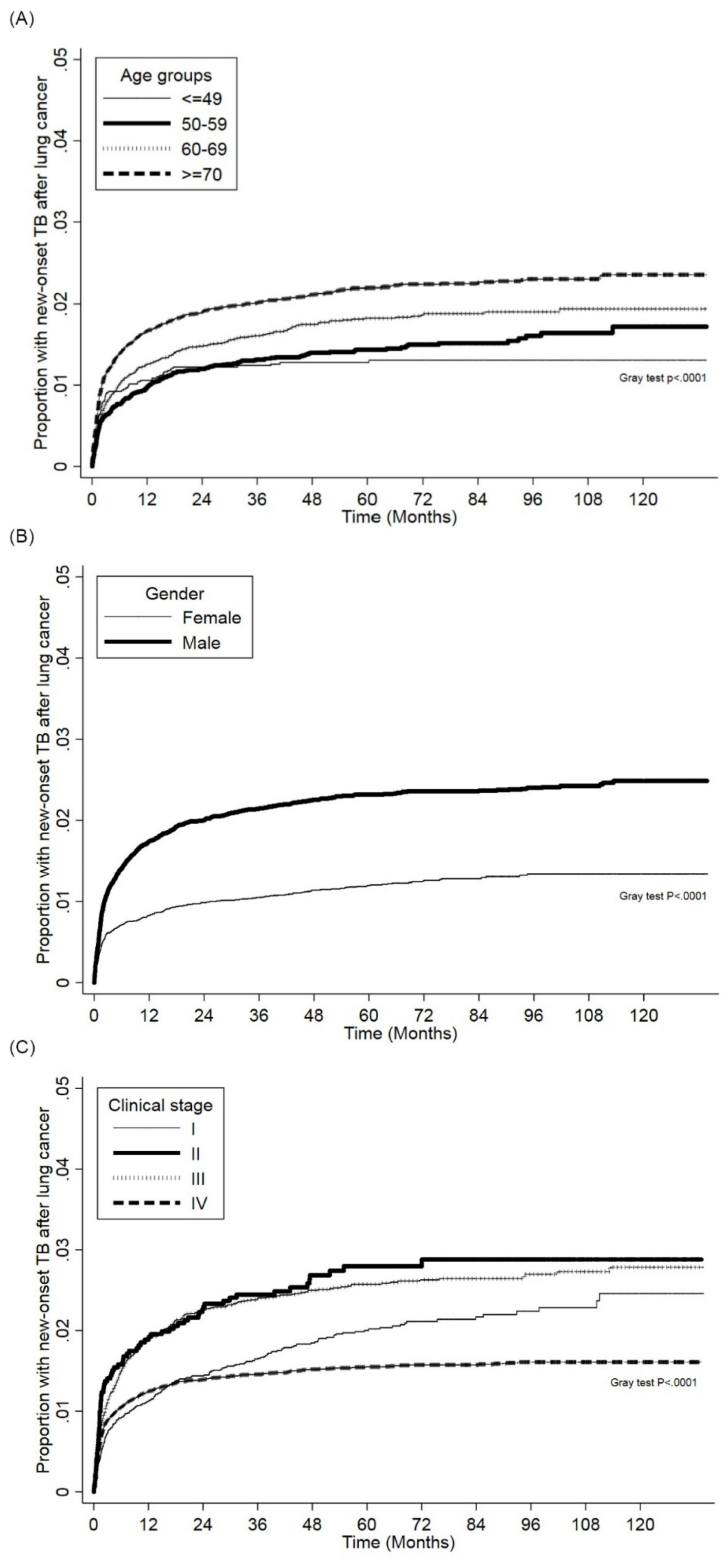
(a). Cumulative incidence function of PTB in patients with lung cancer patients between different age groups. (b). Cumulative incidence function of PTB in patients with lung cancer patients between different sexes. (c). Cumulative incidence function of PTB in patients with lung cancer between different clinical stages.

Figure 2 (a) and Figure 2 (b) show the cumulative incidence of PTB in lung cancer patients of different sexes and age groups, respectively. Males had a significantly higher risk for PTB than females, and the PTB risk increased as patient age increased. The cumulative incidence of PTB in different clinical stages lung cancer patients presented in Figure 2 (c). The PTB risk was lower in stage I compared with other stages.

## Discussion

Our study indicated that patients with lung cancer had the 1.86% of new-onset PTB incidence. The risk factors for PTB in lung cancer patients included male sex, age over 60 years, surgery and chemotherapy, and the comorbidities of pneumoconiosis and hypertension. After further analysis, the PTB risk factors in non-small cell lung cancer patients was male sex, age over 60 years, surgery and chemotherapy, and pneumoconiosis.

PTB and lung cancer are bi-directionally related diseases. There is a causative association between PTB and lung cancer. Previous studies had shown that patients with PTB present an increased risk for lung cancer. Zheng et al. found that patients with PTB history had an approximately 2.5-fold increased risk for lung cancer in a population-based case-control study 9. A single centre study found that patients with a history of PTB had an approximately 11-fold higher risk for lung cancers than those without PTB history 10. The other Taiwan's NHIRD also found the lung cancer incidence was higher in patients with PTB, with an incidence rate ratio of 1.8 (95% CI, 1.3-2.3) 11.

Patients with lung cancer have an increased PTB risk resulting from their immunocompromised status, and lung cancer may enhance PTB infection, reactivate latent PTB infection or cause new exogenous infection 12. Cancer cell destruction of healed PTB lesions may also result in PTB reactivation. Additionally, some factors can deregulate the granuloma microenvironment, such as tumor peptides, antigens, and radiotherapy, allowing PTB bacteria to multiply 13. Risk factors for lung cancer, such as tobacco smoking, COPD, and pneumoconiosis, also increase the risk for PTB. Cigarette smoking was a recognized risk factor for PTB and PTB-related mortality, and our results also presented the significant risk of PTB in lung cancer patients 14, 15. Toki et al. reviewed 442 patients with lung cancer with a mean age of 66 years, and 12.5% patients had coexisting lung cancer and PTB on initial chest X-ray (CXR) when lung cancer was diagnosed; most of the patients had tuberculoma 16. Only one patient with a cavitary lesion had active PTB. Tamura et al. analysed 25 patients with coexisting lung cancer and PTB with a mean age of 70 years at the Tokyo National Chest Hospital from 1991 to 1998 17. The incidence of lung cancer in patients with PTB was 0.7%, while the incidence of PTB in untreated lung cancer patients was 1.9%. Although that study involved data from a single hospital with a limited case number, the incidence of PTB after lung cancer diagnosis in our national registry database was similar. Cha et al. conducted a study at a tertiary referral hospital in Korea and enrolled 36 lung cancer patients; 10 (27.8%) were concomitantly diagnosed with PTB and lung cancer, while 26 (72.2%) were diagnosed with PTB after lung cancer 18. The case number was small, and patients with concomitant PTB and lung cancer were studied. Kamboj et al reported that the incidence of PTB significantly differed according to country of birth and cancer type 19. Foreign-born patients and those with hematologic neoplasms had a risk for PTB approximately 50-100 times higher than US-born patients.

Watanabe et al. 20 found 16 lung cancer patients with coexisting PTB out of 758 overall patients (2.1%), and the cumulative incidence of PTB in different clinical stage of lung cancer were five patients in stage II lung cancer, 3 in stage III, and 8 in stage IV. PTB appeared to be associated with more advanced lung cancer stages. In our study, without consideration of competing risk, PTB incidence was also associated with more advanced cancer stages. However, after adjusting for competing risk, cancer stage and PTB incidence were not significantly associated. The occurrence of mortality in lung cancer patients precluded the occurrence of PTB in our study. Mortality attributable to lung cancer is a competing risk. A patient who dies from cancer is no longer at risk for PTB, regardless of the duration of observation. Analyzing survival data using conventional statistical methods requires the absence of competing risks. When competing risk are present, analysts frequently censor subjects. In our study, PTB incidence had no statistically difference of cancer stage in lung cancer patients with non-small cell or small cell after adjusted with comorbidities and competing with mortality. If competing risks are not considered in the analysis, we found that a significant difference of PTB incidence presented in different clinical stage of lung cancer patients.

Regarding the association between histological subtypes of lung cancer and PTB risk, conflicting results have been reported. Some studies showed that squamous cell carcinoma patients had a higher risk for PTB than patients with other histological subtypes, while some studies showed pulmonary PTB predominance in adenocarcinoma patients 9, 18, 20, 21, 22. Nanthanangkul et al. retrospectively used the Khon Kaen cancer registry and PTB databases to estimate the PTB risk in cancer patients from 2001 to 2015, and PTB infection was common in lung cancer patients but not associated with histopathological type 23. In our study, we divided patients with lung cancer into small cell and non-small cell lung cancer groups, and the risk for PTB was not significantly different between these two groups.

Our study revealed that lung cancer patients who received different treatments did not show the significant risk of PTB; however, after considering competing risk effects of mortality, patients who received operation and chemotherapy had higher risk of PTB than those without receiving operation and chemotherapy, respectively. Jacobs et al. reported a patient with lung cancer with previous PTB history who presented with reactivation of PTB after combined chemotherapy and radiation therapy 24. Thomas et at. described a patient with PTB reactivation after receiving local radiation therapy for prostate cancer 25. Cancer patients are prone to immunosuppression and have increased susceptibility to PTB after chemotherapy, but there are limited studies investigating the risk for PTB after chemotherapy or radiation therapy. It is prudent to check roentgenography and sputum culture after cancer patients undergo chemotherapy because of the impact of this treatment on the immune system. Although the coexistence of lung cancer and PTB was rare, operation was still a treatment choice in lung cancer 26. This may also explain lung cancer patients who receiving operation had high risk of PTB compared with those without in our study.

A review article indicated that recent studies have been conducted on the role of tuberculosis and subsequent risk of lung cancer, but there is limited data about new-onset PTB incidence in patients with lung cancer 27. Therefore, the novelty of this study was that this study definitely examined the risk factor profile of new-onset PTB among overall and different types of lung cancer patients without PTB history using a population real-world database. However, there are some limitations in our study. There was no drug susceptibility testing for *Mycobacterium tuberculosis* in our database. In addition, the disease severity of comorbidities, such as diabetes, ischemic heart disease and renal disease, cannot be obtained from databases. Additionally, patients with lung cancer may not undergo regular sputum culture checks for PTB; thus, PTB may be underdiagnosed in this population.

Our study reveals the incidence rate of PTB among lung cancer patients in a PTB-endemic area. Elderly patients, males, patients who received chemotherapy and patients with a history of pneumoconiosis had a higher risk for PTB if competing risks considered in the analysis. Increased screening for PTB may be necessary among cancer patients with these risk factors to reduce PTB spread and morbidity. A prospective study to delineate the risk for PTB after lung cancer treatment may be needed in the future study.

## Figures and Tables

**Table 1:** Demographic information of lung cancer patients with and without PTB.

Variables	Lung cancer patients		P-value
	Without PTB N (%) = 70,458 (98.14)	With PTB N (%) = 1,335 (1.86)	
**Sex**			
Male	42,219 (97.7)	994 (2.3)	<0.01
Female	28,239 (98.8)	341 (1.2)	
**Age(years)**			
<50	6,531 (98.7)	85 (1.3)	<0.01
50-59	13,494 (98.5)	200 (1.5)	
60-69	17,460 (98.2)	316 (1.9)	
≥70	32,973 (97.8)	734 (2.2)	
**Clinical stage**			
I	11,918 (98.0)	240 (2.0)	<0.01
II	2,837 (97.3)	79 (2.7)	
III	13,343 (97.4)	354 (2.6)	
IV	42,360 (98.5)	662 (1.5)	
**Small cell**	5,714 (98.0)	115 (2.0)	0.50
**Operation, yes**	16,230 (97.7)	377 (2.3)	<0.01
**Chemotherapy, yes**	37,599 (98.0)	783 (2.0)	<0.01
**Radiation therapy, yes**	20,094 (98.1)	386 (1.9)	0.75
**Comorbidity**			
Diabetes	13,529 (98.3)	238 (1.7)	0.21
COPD	11,818 (97.7)	274 (2.3)	<0.01
Renal disease	2,605 (98.4)	43 (1.6)	0.36
Pneumoconiosis	196 (93.3)	14 (6.7)	<0.01
Hypertension	11,602 (98.4)	191 (1.6)	0.04
Dyslipidemia	1,380 (98.8)	17 (1.2)	0.07
Ischemic Heart Disease	2,706 (98.5)	42 (1.5)	0.19
Cerebrovascular Disease	1,439 (98.5)	22 (1.5)	0.31
**Smoking, missing N=30,516**			
No	21,870 (98.8)	261 (1.2)	<0.01
Yes	18,760 (98.0)	386 (2.0)	

**Table 2:** Risk factors for PTB in lung cancer patients.

Variables	Crude HR (95% CI)	P-value	AHR (95% CI)	P-value
**Sex**				
Male vs. Female	2.4(2.1-2.7)	<0.01	2.0(1.7-2.4)	<0.01
**Age(years)**				
<50	Ref		Ref	
50-59	1.1(0.9-1.5)	0.30	1.2(0.9-1.5)	0.27
60-69	1.5(1.2-1.9)	<0.01	1.5(1.2-1.9)	<0.01
≥70	2.3(1.9-2.9)	<0.01	2.2(1.8-2.8)	<0.01
**Clinical stage**				
I	Ref		Ref	
II	1.7(1.3-2.1)	<0.01	1.4(1.1-1.8)	0.02
III	2.0(1.7-2.3)	<0.01	1.5(1.2-1.9)	<0.01
IV	1.5(1.3-1.7)	<0.01	1.4(1.1-1.7)	<0.01
**Small cell**	1.5(1.2-1.8)	<0.01	1.1(0.9-1.4)	0.33
**Operation, yes**	0.7(0.6-0.8)	<0.01	1.1(0.9-1.3)	0.48
**Chemotherapy, yes**	1.2(1.1-1.3)	<0.01	1.0(0.9-1.1)	0.96
**Radiation therapy, yes**	1.1(1.0-1.3)	0.04	1.0(0.9-1.2)	0.74
**Comorbidity**				
Diabetes	1.0(0.9-1.2)	0.96	0.9(0.8-1.1)	0.40
COPD	1.5(1.3-1.7)	<0.01	1.1(0.9-1.2)	0.30
Renal disease	1.1(0.8-1.4)	0.68	0.9(0.7-1.3)	0.70
Pneumoconiosis	4.8(2.9-8.1)	<0.01	2.9(1.7-4.9)	<0.01
Hypertension	0.9(0.8-1.1)	0.35	0.9(0.7-1.0)	0.04
Dyslipidemia	0.6(0.4-1.0)	0.06	0.7(0.4-1.2)	0.17
Ischemic Heart Disease	1.0(0.7-1.3)	0.80	0.9(0.6-1.2)	0.33
Cerebrovascular Disease	1.1(0.7-1.7)	0.58	1.0(0.7-1.6)	0.90
**Smoking, yes**	2.1(1.8-2.5)	<0.01	1.3(1.1-1.5)	<0.01

**Table 3:** The adjusted hazard ratio of PTB derived from the subdistribution hazard ratio and competing mortality risk in lung cancer patients.

Variable	Mortality, %	PTB, %		AHR^ 1^ (95% CI)	P-value
**Sex**					
Male	35,547 (82.3)	994 (2.3)		1.7(1.5-2.0)	<0.01
Female	19,737 (69.1)	341 (1.2)		Ref	
**Age(years)**					
<50	4,439 (67.1)	85 (1.3)		Ref	
50-59	9,211 (67.3)	200 (1.5)		1.2(0.9-1.5)	0.27
60-69	12,673 (71.3)	316 (1.8)		1.4(1.1-1.8)	<0.01
≥70	28,961 (85.9)	734 (2.2)		1.9(1.5-2.4)	<0.01
**Clinical stage**					
I	3,303 (27.2)	240 (2.0)		Ref	
II	1,647 (56.5)	79 (2.7)		1.2(0.9-1.5)	0.20
III	11,064 (80.8)	354 (2.6)		1.2(1.0-1.5)	0.10
IV	39,270 (91.3)	662 (1.5)		0.9(0.7-1.10)	0.24
**Small cell**	5,458 (93.6)	115 (2.0)		0.9(0.8-1.1)	0.44
**Operation, yes**	5,281 (31.8)	377 (2.3)		1.5(1.3-1.8)	<0.01
**Chemotherapy, yes**	32,040 (83.5)	783 (2.0)		1.2(1.1-1.4)	<0.01
**Radiation therapy, yes**	17,981 (87.8)	386 (1.9)		1.0(0.9-1.2)	0.52
**Comorbidity**					
Diabetes	11,195 (81.3)	238 (1.7)		0.9(0.8-1.1)	0.21
COPD	10,052 (83.1)	274 (2.3)		1.0(0.9-1.2)	0.61
Renal disease	2,242 (84.7)	43 (1.6)		0.9(0.6-1.2)	0.29
Pneumoconiosis	180 (85.7)	14 (6.7)		2.6(1.6-4.5)	<0.01
Hypertension	9,677 (82.1)	191 (1.6)		0.9(0.7-1.0)	0.04
Dyslipidemia	1,030 (73.7)	17 (1.2)		0.8(0.5-1.2)	0.27
Ischemic Heart Disease	2,327 (84.7)	42 (1.5)		0.8(0.6-1.1)	0.15
Cerebrovascular Disease	1,328 (90.9)	22 (1.5)		0.9(0.6-1.3)	0.49
**Smoking, yes**	15,217 (79.5)	386 (2.0)		1.2(1.0-1.4)	0.05

1. The adjusted hazard ratio of PTB was derived from the subdistribution hazard ratio and competing mortality risk.

**Table 4:** Risk factors for PTB in patients with lung cancer involving different cell types.

Variable	Non-small cell		Small cell	
	AHR (95% CI)	AHR^1^ (95% CI)	AHR (95% CI)	AHR^1^ (95% CI)
**Sex**				
Male vs. Female	2.0*(1.7-2.3)	1.7*(1.5-2.0)	1.7(0.8-3.6)	1.7(0.8-3.8)
**Age(years)**				
<50	Ref	Ref	Ref	Ref
50-59	1.2(0.9-1.5)	1.2(0.9-1.5)	1.0(0.4-2.5)	1.1(0.4-2.7)
60-69	1.6*(1.2-2.0)	1.5*(1.2-1.9)	0.9(0.4-2.2)	0.9(0.4-2.3)
≥70	2.3*(1.8-2.9)	1.9*(1.5-2.4)	1.5(0.7-3.6)	1.4(0.6-3.3)
**Clinical stage**				
I	Ref	Ref	Ref	Ref
II	1.4*(1.1-1.8)	1.2(0.9-1.6)	1.6(0.3-7.0)	1.2(0.2-6.6)
III	1.6*(1.3-1.9)	1.2(1.0-1.5)	1.1(0.3-3.6)	0.9(0.3-2.9)
IV	1.4*(1.1-1.7)	0.9(0.7-1.1)	1.2(0.4-3.9)	0.7(0.2-2.4)
**Operation**	1.1(0.9-1.3)	1.6*(1.3-1.9)	0.5(0.1-1.9)	0.6(0.1-2.2)
**Chemotherapy**	1.0(0.9-1.1)	1.2*(1.0-1.3)	1.3(0.8-2.4)	2.4*(1.4-4.4)
**Radiation**	1.0(0.9-1.2)	1.0(0.9-1.2)	0.9(0.6-1.3)	1.1(0.7-1.6)
**Comorbidity**				
Diabetes	0.9(0.8-1.1)	0.9(0.8-1.0)	1.2(0.8-1.8)	1.1(0.7-1.7)
COPD	1.1(0.9-1.2)	1.0(0.9-1.2)	1.2(0.8-1.8)	1.1(0.7-1.7)
Renal disease	1.0(0.7-1.4)	0.9(0.7-1.2)	0.6(0.2-1.9)	0.4(0.1-1.6)
**Pneumoconiosis**	3.3*(2.0-5.6)	3.0*(1.8-5.1)	0.9(0.1-15.7)	-
Hypertension	0.9(0.7-1.0)	0.8(0.7-1.0)	0.9(0.5-1.5)	0.9(0.5-1.5)
Dyslipidemia	0.6(0.4-1.1)	0.7(0.4-1.1)	1.9(0.6-5.7)	1.8(0.5-6.3)
Ischemic Heart Disease	0.9(0.6-1.2)	0.8(0.6-1.1)	0.7(0.3-2.0)	0.6(0.2-2.0)
Cerebrovascular Disease	1.1(0.7-1.7)	0.9(0.6-1.4)	0.3(<0.01-4.1)	-
**Smoking, yes**	1.3*(1.1-1.5)	1.2(1.00-1.4)	1.1(0.5-2.5)	1.2(0.5-2.8)

1. The adjusted hazard ratio of PTB was derived from the subdistribution hazard ratio and competing mortality risk. *. P-value less than 0.05.

## Abbreviations

PTB: Pulmonary tuberculosis infection
TCR: Taiwan Cancer Registry
NHIRD: Taiwan's National Health Insurance Research Database
HWDC: Health and Welfare Data Science Center
ICD-CM: International Classification of Diseases, Clinical Modification codes
ATC: Anatomical Therapeutic Chemical
COPD: Chronic obstructive pulmonary disease
CI: Confidence interval
